# Glycocalyx-Shedding and Inflammatory Reactions Occur Yet Do Not Predict Complications Resulting from an Esophagectomy in an Accelerated Recovery After Surgery Program

**DOI:** 10.3390/jcm14176048

**Published:** 2025-08-26

**Authors:** Hendrik Drinhaus, Christoph Mallmann, Corvin Cleff, Tobias Neumann, Christina Daniels, Christiane J. Bruns, Andrea U. Steinbicker, Wolfgang Schröder, Thorsten Annecke

**Affiliations:** 1 Department of Anaesthesiology and Intensive Care Medicine, Faculty of Medicine, University Hospital of Cologne, University of Cologne, 50937 Cologne, Germany; corcleff@web.de (C.C.); neumann@familienmedizin.koeln (T.N.); christina.daniels@evkk.de (C.D.); andrea.steinbicker@uk-koeln.de (A.U.S.); annecket@kliniken-koeln.de (T.A.); 2 Department of General, Visceral, Cancer and Transplantation Surgery, Faculty of Medicine, University Hospital of Cologne, University of Cologne, 50937 Cologne, Germany; christoph.mallmann@helios-gesundheit.de (C.M.); christiane.bruns@uk-koeln.de (C.J.B.); wolfgang.schroeder@helios-gesundheit.de (W.S.); 3 Department of General, Visceral and Oncological Surgery, Helios University Hospital Wuppertal, University of Witten/Herdecke, 58455 Witten, Germany; 4 Private Practice for Family Medicine familienmedizin.koeln, 50829 Cologne, Germany; 5 Cologne Merheim Medical Center, Department of Anaesthesiology and Intensive Care Medicine, University of Witten/Herdecke, 58455 Witten, Germany

**Keywords:** esophagectomy, esophageal cancer, endothelial glycocalyx, CO_2_-gap, Interleukins, enhanced recovery after surgery, perioperative complications

## Abstract

**Background/Objectives:** “Accelerated Recovery after Surgery” (ARAS) programs for esophagectomy aim to shorten the perioperative course without increases in morbidity or mortality. In such programs, the prediction and early detection of perioperative complications is essential, as ICU observation times are limited. We evaluated two potential laboratory markers as predictors for postoperative complications: shedding of the endothelial glycocalyx and the veno-arterial CO_2_-gap as indicators of microcirculatory disturbances. **Methods:** In total, 26 patients undergoing hybrid Ivor Lewis esophagectomy within an ARAS program were included. Macrocirculatory conditions were kept stable by enhanced hemodynamic monitoring (PiCCO). Glycocalyx shedding parameters (Syndecan-1, heparan sulfate, hyaluronic acid) and a panel of inflammatory mediators were measured preoperatively, upon ICU-admission, and on the first postoperative day. The veno-arterial CO_2_-gap was calculated at induction of anesthesia, during laparoscopy, and upon admission to the ICU. **Results:** Complications (Dindo-Clavien ≥3) occurred in *n* = 16 (62%) patients. From preoperatively to admission to the ICU, Syndecan-1 (29 pre-op to 56 ng/mL at ICU-admission) and Interleukins 1b (1.2 to 1.4 pg/mL), 6 (1.3 to 19.9 pg/mL), 8 (5.2 to 19.9 pg/mL), and 10 (0.50 to 1.33 pg/mL) increased, indicating a temporary increase in inflammation and glycocalyx shedding during surgery. A difference between patients with or without complications could not be detected. There was also no difference in the veno-arterial CO_2_-gap between the two groups (median of 6.8 mmHg in all patients, 6.7 in patients with complications, 7.8 in patients without complications). **Conclusions:** Signs of microcirculatory dysfunctions and inflammation occurred during esophagectomy within an ARAS protocol with tightly controlled hemodynamics. Increases in Syndecan-1 and the veno-arterial CO_2_-gap could not predict perioperative complications.

## 1. Introduction

Esophagectomy is a high-risk surgical procedure as defined by the guidelines for preoperative risk evaluation of the European Society of Cardiology and the European Society of Anesthesiology [1], independent of any individual patient’s additional risk factors. Particularly, transthoracic esophagectomy with intrathoracic anastomosis of a gastric sleeve (Ivor Lewis esophagectomy) carries a high perioperative risk, despite its long-term advantages compared with transhiatal esophagectomy [2]. An association between experience and caseload and outcome has been shown; the operation is therefore recommended to be performed in high-volume centers. Therefore, complications do not necessarily occur less often, but inadequate management of complications (“failure to rescue”) with adverse clinical outcomes seems to be less common [3,4,5,6,7]. Hence, even if complications such as anastomotic leakage occur with the same frequency, their impact on mortality and morbidity is less profound when treated early and adequately. Accelerated recovery after surgery (ARAS), also termed enhanced recovery after surgery (ERAS^®^) or fast track programs, have been established to fasten perioperative treatment courses while maintaining low complication rates or even further reducing complications [8,9]. Due to reduced postoperative observation times, the timely detection and treatment of complications is particularly important. New predictors of perioperative complications would be valuable for this purpose. Even if the macrocirculation remains closely monitored and controlled within such programs, intraoperative disturbance of the microcirculation might occur and precede postoperative complications. In this context, the vascular endothelial glycocalyx, a structure of proteoglycans, glycosaminoglycans, and glycoproteins lining the vascular wall, in association with plasma contents (“endothelial surface layer”), may be an interesting target for diagnostic and therapeutic interventions. Soluble glycocalyx components released during microcirculatory disturbances might serve as biomarkers and predictors of early postoperative complications. The endothelial surface layer plays a major role in several functions of the vasculature, such as maintenance of vascular barrier, mechano-transduction of shear stress, and leucocyte–endothelium interaction. Destruction of the glycocalyx and shedding of its components into the blood occur early in a variety of pathologies, including shock, major trauma or surgery, cardiopulmonary resuscitation, ischemia/reperfusion, or chronic metabolic or cardiovascular diseases [10,11,12,13,14]. Hypervolemia might also contribute to glycocalyx shedding [15]. In a study of patients undergoing abdominal surgery, patients suffering from sepsis had a higher Syndecan-1 concentration in blood on the first postoperative day than non-septic patients [16]. Recently, elevated Syndecan-1 levels (>48 ng/mL) after robot-assisted McKeown esophagectomy have been shown to be associated with morbidity and mortality [17].

An increased (e.g., >6 mmHg) difference in carbon dioxide tension or content between mixed or central venous and arterial blood (the so-called CO_2_-gap) has been described as a potential marker of impaired microcirculation and tissue hypoperfusion during shock states and after major abdominal surgery [18,19,20].

We hypothesized that a high CO_2_-gap as a sign of disturbances of the microcirculation and an increased glycocalyx-shedding within the “prediction window” from induction of anesthesia to the first postoperative day precedes postoperative complications within a complication observation period of 14 days in a setting of strictly controlled and maintained macrocirculatory hemodynamics and fluid status.

## 2. Materials and Methods

### 2.1. Ethical Approval and Study Registration

Ethical approval was obtained from the ethics committee of the Medical Faculty of the University of Cologne (approval number 15-409, approval date 18 December 2015). All patients signed informed consent forms. The trial was registered at the German Registry of Clinical Trials (DRKS00009513), and details of the study protocol have been published earlier [21].

### 2.2. Patient Selection

In total, 26 patients undergoing hybrid minimally invasive Ivor Lewis esophagectomy with laparoscopic preparation of the stomach for squamous cell carcinoma or adenocarcinoma of the esophagus between 2015 and 2018 were included in this prospective investigation. The inclusion criteria were ASA-category I or II body mass index 20–30 kg/m^2^, preoperative weight loss <10% in 3 months, forced expiratory volume in one second (FEV_1_) >80% of predicted value, and perioperative availability of a trained member of the study team. The exclusion criteria were a history of myocardial infarction, stroke, peripheral artery disease > stage IIa, estimated glomerular filtration rate <60 mL/min, or insulin-treated diabetes. The ARAS protocol was aborted, and patients were excluded from the study in case of inability to insert the epidural or arterial (PiCCO-system) catheter, conversion to laparotomy, extubation >4 h postoperatively, or ICU-stay >48 h. Glycocalyx and CO_2_-gap data were missing from two patients, inflammation data were missing from one patient, while demographic and clinical data could be reported for 26 patients, data on glycocalyx shedding and the CO_2_-gap could be analyzed for 24 patients, and inflammation data could be analyzed for 25 patients.

### 2.3. ARAS Protocol

Patients underwent an accelerated recovery after surgery (ARAS) program, including physiotherapy, intake of carbohydrate drinks until two hours before surgery and early start of postoperative enteral feeding, thoracic epidural anesthesia, no sedative premedication and extubation in the operating theatre, perioperative hemodynamic monitoring, early removal of the chest tube, and routine endoscopy on postoperative day 5 (Table 1) [21,22]. As our program, which we termed “accelerated recovery after surgery (ARAS),” was devised and taken out before publication of the guidelines of the ERAS^®^-society on esophagectomy [8], it is not completely identical with the ERAS^®^-program and thus received a separate name: ARAS.

### 2.4. Hemodynamic Protocol

Hemodynamic parameters were measured using standard anesthesia monitoring and pulse contour analysis and transpulmonary thermodilution with the PiCCO Plus-system (Getinge/Maquet). The basal rate of crystalloid infusion was set at 2 mL/kg/predicted body weight per hour. Thermodilution measurements were performed in the operating theatre (1) after induction of anesthesia, (2) after primary hemodynamic optimization, (3) after beginning of the laparoscopic part of the operation, (4) after beginning of the thoracic part of the operation, (5) after induction of one-lung-ventilation, (6) after completion of the operation and (7) on the intensive care unit (ICU) upon admission, and (8) at midnight of the first postoperative night and every morning thereafter during the ICU-stay.

Hemodynamic targets were cardiac index >2.5 L/min/m^2^, stroke volume variation <12%, and a systemic vascular resistance index (SVRI) of 1200–2000 dyn. Additional crystalloid boluses (3 mL/kg), vasopressor (Norepinephrine infusion), or inotropic (Dobutamine infusion) therapy were used as needed to reach these targets. No colloid infusions were applied.

### 2.5. Measurement of Glycocalyx Shedding

For the detection of glycocalyx shedding, typical soluble components of this structure were measured in plasma at different time points. Blood samples (Sarstedt S-Monovette, Sarstedt, Nümbrecht, Germany) were drawn (1) after induction of anesthesia, (2) upon admission to the ICU, and on the morning of the (3) first postoperative day. Plasma samples were centrifuged, divided into aliquots (to avoid freeze–thaw cycles), and frozen at −80 °C until further analysis. Glycocalyx shedding was measured as previously described [14]: ELISA was used to measure concentrations of Syndecan-1, heparan sulfate, and hyaluronic acid. In total, 50–100 µL blood samples or standard concentrations supplied by the manufacturer (Syndecan-1, Diaclone, Besancon, France; heparan sulfate, Cusabio Biotech CO., Wuhan, China; Hyaluronic acid, Echelon Biosciences, Salt Lake City, UT, USA) were applied to antibody-coated wells and reagents added, washed sequentially as prescribed. Adsorption of light at the respective wavelength was measured in a 96-well microplate reader (Multiskan GO, Thermo Fisher Scientific, Waltham, MA, USA). We calculated concentrations of the respective glycocalyx components with Microsoft Excel by using a standard curve method derived from the standards supplied by the manufacturer. Precision-, Linearity-, and Spike-and-recovery-test have been performed by the manufacturers of the ELISA kits, and according to the manufacturer, there is no cross-reactivity between heparan sulphate and heparin.

### 2.6. Measurement of Inflammation Markers

Levels of Interleukin 1b (IL-1b), Interleukin 6 (IL-6), Interleukin 8 (IL-8), Interleukin 10 (IL-10), Interleukin 12 dimer (IL-12 p70), and tumor necrosis factor (TNF) obtained at the same time points as described above were measured using Human Inflammatory Cytokine CBA Kit (551811, BD Biosciences) according to the manufacturer’s guidelines. After acquisition of sample data using fluorescence-activated cell sorting (FACS Calibur; BD Bioscience), concentrations were calculated using the proprietary FCAP Version 1.0 analysis software (BD Biosciences, Franklin Lakes, NJ, USA). Precision-, Linearity-, and Spike-and-recovery-test have been performed by the manufacturer of the CBA-kit.

### 2.7. Measurement of the Veno-Arterial CO_2_-Difference in Blood

Blood was drawn simultaneously from an arterial and central venous line and analyzed in a Radiometer ABL 800 Analyzer. CO_2_ values were not corrected for temperature and the veno-arterial CO_2_-Gap was calculated by subtracting paCO_2_ from pvCO_2_.

### 2.8. Grading of Perioperative Complications

Perioperative complications were graded according to the Dindo-Clavien classification of surgical complications [23]. An uncomplicated perioperative course was defined as no complications or Grade I or II complications. A complicated perioperative course was defined as Grade III to V depending on the degree of complications.

### 2.9. Statistical Analysis

Data were registered and processed with Microsoft Excel v 16.0 (Microsoft, Redmond, WA, USA). Statistical analyses were performed with R and GraphPad Prism 10.3.1. Visualizations were created with GraphPad Prism 10.3.1 (GraphPad Software, Boston, MA, USA). Most of the data were not normally distributed. For consistency, we used median and interquartile range for all descriptive statistics and hypothesis testing. The Mann–Whitney U test was used to test for inter-group differences with ordinal data, the Wilcoxon signed-rank test was used for testing of paired ordinal data, and Fisher’s exact test was used for testing of categorical data. The statistical significance threshold was defined as *p* < 0.05.

### 2.10. Use of Generative Artificial Intelligence

No Generative Artificial Intelligence has been used for the preparation of this study.

## 3. Results

### 3.1. Patients’ Characteristics

Patients’ characteristics are listed in Table 2. In 16 of 26 patients, complications of Dindo-Clavien Grade > II occurred. The Comprehensive Complications Index (CCI^®^) [24] score of the patients with complications Dindo-Clavien Grade > II ranged between 26.2 and 56.3, whereas the CCI of patients with Dindo-Clavien Grade ≤ II ranged from 0 to 20.9. Hospital stay was longer in patients with complications (median 16 days, interquartile range 11–23 days) than those without (11 days, IQR 10–12 days, *p* = 0.032). The spectrum of clinical complications included delayed gastric conduit emptying (11/26, 42.3%), anastomotic leakage (2/26, 7.7%), atrial fibrillation (2/26, 7.7%), pneumonia (2/26, 7.7%), pleural effusion with requiring new pleural drainage (1/26, 3.8%), and reintubation and tracheostomy (1/26, 3.8%).

### 3.2. Glycocalyx Shedding

**Figure 1:**
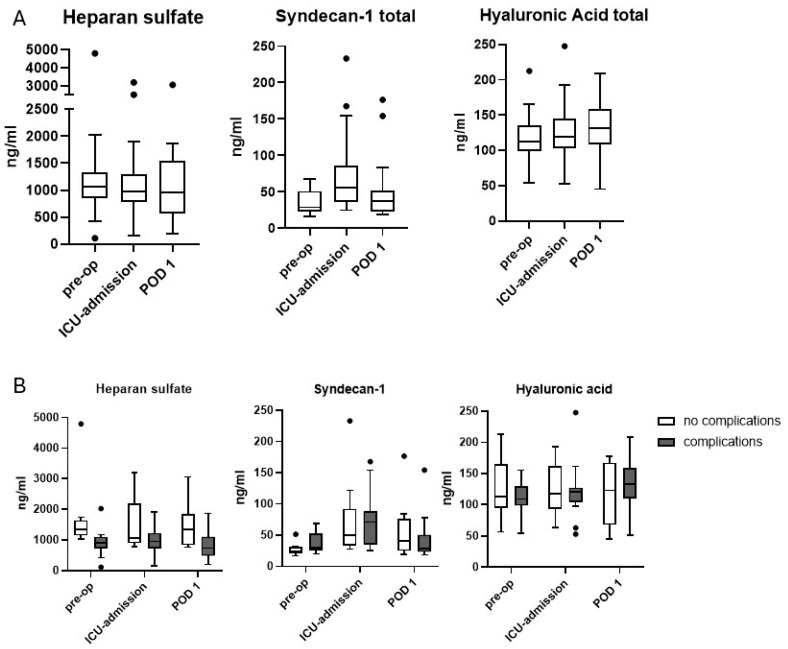
Levels of the glycocalyx components heparan sulfate, syndecan-1, and hyaluronic acid in blood serum. (**A**): values of the entire study population. (**B**): values separated according to complication status. Box plot, Tukey method. Dots represent outliers above the 75th percentile plus 1.5 interquartile ranges in the Tukey method.

Heparan sulfate concentrations did not increase during the perioperative course. Preoperatively, levels of heparan sulfate were lower in patients with developed perioperative complications (901 ng/mL, IQR 727–1098 ng/mL) than in those who did not develop complications (1343 ng/mL, IQR 1146–1634 ng/mL) (Figure 1 and Table 3).

Syndecan-1 levels increased significantly (*p* < 0.01 for the difference between time point 1 and 2) and approximately doubled during surgery in both groups. During the ICU stay, we observed a reduction, with a decrease to preoperative levels at day 2. There were no statistically significant differences in Syndecan-1 levels between the groups of patients with or without complications (Figure 1 and Table 3). No relevant increase was detected in hyaluronic acid levels within the observation period (Figure 1 and Table 3).

### 3.3. Interleukins and Tumor Necrosis Factor

The concentrations of Interleukin 1b, Interleukin 6, Interleukin 8, and Interleukin 10 increased during surgery, whereas no changes in Interleukin 12p70 and tumor necrosis factor alpha occurred. Apart from a slightly higher level of Interleukin 10 in the complications group on the first postoperative day, no differences were detected between patients with or without complications (Table 4).

### 3.4. CO_2_-Gap at Admission to the Intensive Care Unit

**Figure 2:**
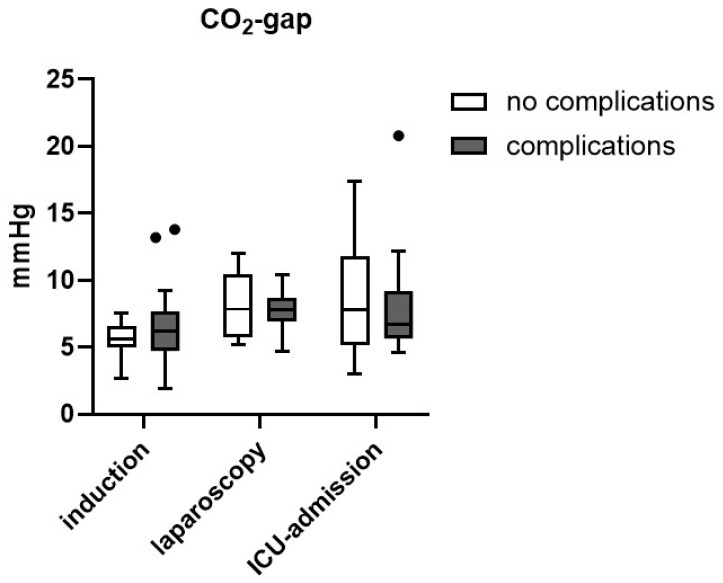
Veno-arterial CO2-gap in patients with or without complications. Box plot, Tukey method. Dots represent outliers above the 75th percentile plus 1.5 interquartile ranges in the Tukey method.

The median CO_2_-gap (difference between central venous and arterial pressure of carbon dioxide) increased between induction of anesthesia and the laparoscopic part of surgery from 5.7 mmHg (IQR 4.9–7.1 mmHg) to 7.8 mmHg (IQR 6.4–9.6 mmHg) (* *p* = 0.049). The median CO_2_-gap at admission to the intensive care unit was 6.8 mmHg (IQR 5.6–10.4 mmHg) overall. In patients with complications, the median CO_2_-gap at ICU-admission was 6.7 mmHg (IQR 5.7–9.2 mmHg), in those without complications, 7.8 mmHg (IQR 5.2–11.8 mmHg), the difference between both groups was not significant (Figure 2).

## 4. Discussion

In this study of a small cohort of selected patients undergoing hybrid minimally invasive Ivor Lewis esophagectomy with enhanced hemodynamic monitoring within an ARAS program, we observed a perioperative increase in Syndecan-1 shedding as well as an increase in the concentrations of IL-6, IL-8, and IL-10. We could not confirm our hypothesis that glycocalyx shedding is more pronounced in patients suffering from perioperative complications than in those without. Hence, our data do not suggest the use of glycocalyx-shedding parameters as early indicators of perioperative complications after Ivor Lewis esophagectomy within an ARAS program. The veno-arterial difference in carbon dioxide pressure (“CO_2_-gap”) was high in all patients but not significantly different in patients with or without perioperative complications. Using a standardized hemodynamic treatment algorithm, we have minimized intravascular volume disturbances as potential confounders affecting glycocalyx shedding. Glycocalyx shedding, as expressed by a release of Syndecan-1 into the systemic circulation, did occur, despite careful preservation of a normal macrocirculatory circulation and strict avoidance of fluid overload.

As no studies analyzing glycocalyx shedding after hybrid Ivor Lewis esophagectomy have been published in MEDLINE-listed journals hitherto, our findings have to be discussed in the context of prior studies with different surgical techniques (and hence different amounts of surgical trauma) for esophagectomy. A recent investigation of *n* = 207 patients undergoing robot-assisted McKeown esophagectomy found that a postoperative Syndecan-1 level of ≥48 ng/mL was associated with a composite endpoint of 30-day mortality and different measures of perioperative morbidity [17]. Another study in *n* = 112 patients undergoing major abdominal surgery investigated an increase in Syndecan-1 (from 17 to 46 ng/mL) and IL-6 (from 0 to 84 pg/mL) perioperatively but could not detect an association between perioperative morbidity (Comprehensive Complication Index ≥ 26.2) and increased Syndecan-1 levels. In total, 45 of the 112 cases analyzed in that study were open Ivor Lewis esophagectomies, but a subgroup analysis for this kind of surgery is not available [25]. In an analysis of the correlation between Syndecan-1 levels and sepsis in 55 patients after major abdominal surgery, patients suffering from sepsis had higher levels of Syndecan-1 (90 ng/mL) than those without sepsis (17 ng/mL) on the first postoperative day. Preoperatively and directly after surgery, there were no differences between patients with and without the development of sepsis [16]. In our study, mean preoperative baseline levels of Syndecan-1 were already higher than 20 ng/mL. Syndecan-1 increased significantly to more than 60 ng/mL after surgery, also in the group of patients without major complications. Syndecan-1 and hyaluronic acid levels in blood were analyzed in a study of *n* = 60 patients undergoing lung resection. Syndecan-1 levels increased slightly but significantly during surgery; whereas, in our study, no significant perioperative increase in hyaluronic acid was detected. In that study, baseline levels of both soluble glycocalyx components (syndecan-1 and hyaluronic acid) were much lower than in our patients, too [26]. Another study compared the effect of sevoflurane or propofol on shedding of heparan sulfate and Syndecan-1 during one-lung ventilation and reported lower baseline and postoperative levels of both glycocalyx components compared to our results [27]. This might allude to the elevation of Syndecan-1 levels in patients with esophageal cancer even without surgery, as we already detected in patients with cancers of the oral cavity [14]. Hence, it remains to be elucidated in larger trials whether patients with esophageal cancer, who have undergone radiochemotherapy in most cases, in general exhibit higher baseline levels of glycocalyx components in the blood. A potential contribution of neoadjuvant chemoradiotherapy has not been investigated yet, but the fact that neoadjuvant chemoradiotherapy was associated with high syndecan-1 levels in patients undergoing McKeown esophagectomy might allude to this possibility [17]. For urogenital cancers, high local or systemic concentrations of Syndecan-1 have been shown [28,29]. Alterations of Syndecan-1 expression in the tumor stroma have also been suggested for squamous cell carcinoma of the esophagus [30]. Furthermore, Heparanase, an enzyme linked to shedding of the glycocalyx, appears to be upregulated in Barrett‘s esophagus, a precursor of adenocarcinoma of the esophagus [31]. However, healthy volunteers tested in our laboratory exhibited similar seemingly high levels of Syndecan-1 in blood (Syndecan-1 29 ng/mL, heparan sulfate 1329 ng/mL, hyaluronic acid 92 ng/mL [median]) [32]. Whether the higher preoperative value of heparan sulfate in patients without complications has a pathophysiological foundation or whether it was caused artificially by a single exceedingly high outlying value (cf. Figure 1b) remains to be verified in further studies.

The different time course of changes in the respective glycocalyx components is in accordance with a recent study on glycocalyx shedding after cardiac arrest [12], in which a correlation between shedding of Syndecan-1 and hyaluronic acid and death was observed. Syndecan-1 levels peaked 24 h after cardiac arrest, whereas hyaluronic acid levels peaked only after 48 h. In our study, the highest Syndecan-1 levels occurred immediately after surgery, whereas a small increase in hyaluronic acid was only detected later.

Interleukin 6 concentrations increased during surgery, peaked upon admission to the ICU, and started decreasing on postoperative day 1. In a study of *n* = 97 patients undergoing esophagectomy, interleukin-6 was found to be increased more in patients developing pulmonary complications at later time points. Albeit with a different time course, those results are in line with our study [33]. Another trial compared conventional and fast-track (in some parts similar to our ARAS protocol) processes for esophagectomy and found slightly reduced levels of interleukin-6 in the fast-track group [34]. In line with this, in another trial, patients with epidural analgesia showed less interleukin-6 increase after esophagectomy than those without epidural analgesia [35]. An intriguing finding in our study is the “borderline-significant” (*p* = 0.08) higher level of the anti-inflammatory cytokine Interleukin 10 on the first postoperative day in the complications group. This should be studied further in larger trials.

The difference between venous and arterial pressure of carbon dioxide, the so-called CO_2_-gap, has been described as a marker of inadequate tissue perfusion, reflecting disturbances in the microcirculation, in recent years. Some studies have found increased CO_2_-gaps in patients with postoperative complications after cardiac and non-cardiac surgery. Different cut-off values, ranging from 5 to 8 mmHg, have been proposed [20]. We did not detect differences in the CO_2_-gap upon admission to the intensive care unit after transthoracic esophagectomy in patients with or without complications. In both groups, the CO_2_-gap was high (>7 mmHg) and above the cut-off value described as indicative of postoperative complications in other trials. Together with the increase in soluble Syndecan-1 as a marker of glycocalyx-shedding, the high CO_2_-gap may be a sign of a “decoupling” between micro- and macrocirculation in the setting of esophageal surgery despite strict control of adjustable macrocirculatory parameters. The finding that the veno-arterial gap in CO_2_-pressure is not a suitable predictor of perioperative complications is in line with a recent study that investigated the CO_2_-gap in a sample of 464 patients undergoing major abdominal surgery (*n* = 76 were esophageal resections), where a median CO_2_-gap of 6.0 mmHg was found in both groups-with and without complications [36].

### Limitations

The main limitation of our study is its monocentric nature and the small number of patients included. To corroborate our conclusions, a future multicentric study including more patients would be advisable. In addition, our patient cohort was deliberately highly selected, so the external validity of the findings of the present study to more heterogeneous patient collectives without tightly controlled study programs remains to be verified in patient collectives with more generous inclusion and exclusion criteria. Also, glycocalyx shedding was only investigated by measurements of soluble components of the endothelial glycocalyx in the systemic circulation. Other methods for the detection of glycocalyx shedding, such as sublingual intravital microscopy [32,37], might provide additional insights into alterations of the endothelial surface layer during esophagectomy but must be used with caution in the clinical context of esophagectomy. Due to its limitations, this study should be regarded as a “pilot-study”, stipulating further research on the topic in larger, more heterogeneous “real-world” patient collectives to gain more statistical power and external validity, ideally using multimodal analysis of the endothelial glycocalyx/endothelial surface layer with measurement of glycocalyx components in blood and intravital microscopy.

## 5. Conclusions

In this sample of highly selected patients undergoing transthoracic esophagectomy with enhanced hemodynamic monitoring to minimize the potential confounder of fluid imbalances, we detected perioperative shedding of the glycocalyx component Syndecan-1 but not heparan sulfate or hyaluronic acid. An increase in IL-1b, -6, -8, and -10 and the veno-arterial CO_2_-gap was present in all patients. Therefore, microcirculatory disturbance may occur despite strict control of macrocirculatory parameters during this major surgical procedure. However, we could not identify glycocalyx shedding or the veno-arterial CO_2_-gap as reliable biomarkers for the early identification and discrimination of patients with or without perioperative complications. Furthermore, larger trials should corroborate the findings of this “pilot study”.

## Figures and Tables

**Table 1:** Main features of the ARAS protocol (modified from [20,21]). POD: postoperative day.

nutrition	Small portions of water from POD1 Enteral feeding completed at POD7 Supportive parenteral nutrition up to POD4
anesthesia/surgery management	no routine benzodiazepine premedication Extubation in the operating theatre Restrictive volume management based on hemodynamic monitoring hybrid- or total minimally invasive Ivor Lewis esophagectomy Discharge from ICU on POD 1 (if clinically stable)
Physiotherapy/Mobilization	Mobilization and physiotherapy from operating day on Physiotherapy training twice daily
Endoscopy	Routine endoscopy and dilatation of the pylorus (if necessary) on POD5
Chest drain/nasogastric tube	Removal of chest drain if <300 mL/24h from POD1 on Removal of chest drain on POD 5 at the latest No nasogastric tube
Discharge from the hospital	POD 10

**Table 2:** Patient characteristics of patients in both groups. IQR: interquartile range MAP: mean arterial pressure. Median + IQR (except for categorical variables).

	Overall (*n* = 26)	No Complications (*n* = 10)	Complications (*n* = 16)	*p*-Value
Age [years]	55 [51; 60]	57 [53; 64]	54 [50; 56]	0.11
Sex (female/male)	3/23	2/8	1/15	0.54
ASA class (I/II)	13/13	5/5	8/8	>0.999
CCI	26.2 [0; 26.2]	0.0 [0.0; 0.0]	26.2 [26.2; 31.8]	<0.0001
Body mass index [kg/m^2^]	25 [23; 28]	24 [23; 27]	25.5 [24; 28]	0.54
Adenocarcinoma/squamous cell carcinoma	22/4	9/1	13/3	>0.999
Duration of surgery [min]	340 [314; 354]	345 [336; 351]	336.5 [310; 363]	0.71
Maximal Norepinephrine requirement [µg/kg/min]	0.06 [0.05; 0.1]	0.06 [0.05; 0.11]	0.05 [0.05; 0.08]	0.28
Cardiac Index upon admission to the ICU [L/min/m^2^)	3.4 [3.3; 3.5]	3.2 [2.5; 3.6]	3.5 [3.1; 4.0]	0.26
Lowest Cardiac Index	2.9 [2.8; 3.5]	2.8 [2.5; 3.6]	2.9 [2.6; 3.4]	0.34
Lowest intraoperative MAP [mm Hg]	68 [65; 73]	66 [64; 70]	70 [65.25; 73]	0.30
Intraoperative fluid balance [L]	+1.2 [0.8; 1.6]	+1.1 [0.9; 1.4]	+1.2 [0.7; 1.6]	0.71
Fluid balance after 24 h [L]	+1.5 [−0.2; +2.0]	+1.8 [0; +2.2]	+1.4 [−0.2; 1.8]	0.72

**Table 3:** Levels of glycocalyx components and (median + IQR) in patients with or without complications at different time points.

	Overall (*n* = 24)	No Complications (*n* = 9)	Complications (*n* = 15)	*p*-Value
Heparan sulfate [ng/mL]				
- Pre-operative	1086 [857; 1334]	1343 [1146; 1634]	901 [727; 1098]	0.001
- ICU-admission	982 [781; 1292]	1062 [898; 2175]	948 [726; 1225]	0.073
- POD 1	952 [566; 1540]	1351 [829; 1832]	727 [487; 1107]	0.034
Syndecan-1 [ng/mL]				
- Pre-operative	29 [23; 51]	24 [21; 31]	30 [26; 53]	0.084
- ICU-admission	56 [36; 87]	50 [33; 62]	71 [35; 88]	0.60
- POD 1	37 [23; 52]	40 [25; 76]	28 [23; 50]	0.59
Hyaluronic acid [ng/mL]				
- Pre-operative	113 [99; 136]	113 [95; 164]	109 [99; 129]	0.32
- ICU-admission	119 [103; 145]	117 [93; 162]	121 [104; 127]	0.95
- POD 1	131 [108; 159]	123 [68; 167]	133 [110; 159]	0.47

**Table 4:** Levels of interleukins and TNF (median + IQR) in patients with or without complications at different time points.

	Overall (*n* = 25)	No Complications (*n* = 9)	Complications (*n* = 16)	*p*-Value
Interleukin 1b [pg/mL]				
- Pre-operative	1.2 [0.9; 1.7]	1.3 [0.9; 1.5]	1.1 [0.8; 2,1]	0.97
- ICU-admission	1.4 [1.0; 2.5]	1.3 [1.1; 2.2]	1.6 [1.0; 2.9]	0.56
- POD 1	1.2 [0.9; 2.5]	1.2 [1.0; 2.2]	1.2 [0.8; 3.2]	0.98
Interleukin 6 [pg/mL]				
- Pre-operative	1.7 [1.3; 3.2]	2.5 [1.5; 3.7]	1.7 [1.1; 2.9]	0.35
- ICU-admission	177.0 [100.8; 251.0]	194.5 [111.4; 379.1]	151.1 [97.1; 233.9]	0.30
- POD 1	87.2 [54.2; 140.3]	99.3 [65.4; 147.6]	86.0 [34.8; 136.9]	0.38
Interleukin 8 [pg/mL]				
- Pre-operative	5.2 [3.6; 8.2]	5.2 [3.5; 7.0]	5.2 [3.3; 9.6]	0.88
- ICU-admission	19.9 [13.0; 32.9]	21.5 [18.5; 33.8]	19.1 [11.3; 33.1]	0.60
- POD 1	20.7 [13.6; 26.6]	23.6 [15.1; 36.5]	19.2 [11.8; 23.9]	0.35
Interleukin 10 [pg/mL]				
- Pre-operative	0.50 [0.45; 0.61]	0.50 [0.43; 0.65]	0.51 [0.45; 0.59]	0.94
- ICU-admission	1.33 [0.84; 3.54]	1.50 [1.04; 2.09]	0.98 [0.81; 3.93]	0.79
- POD 1	0.73 [0.64; 1.15]	0.67 [0.63; 0.73]	0.86 [0.65; 1.26]	0.08
Interleukin 12 [pg/mL]				
- Pre-operative	0.42 [0.30; 0.54]	0.49 [0.39; 0.55]	0.33 [0.22; 0.54]	0.13
- ICU-admission	0.39 [0.32; 0.61]	0.37 [0.35; 0.43]	0.42 [0.27; 0.82]	0.48
- POD 1	0.33 [0.27; 0.44]	0.32 [0.29; 0.41]	0.36 [0.26; 0.47]	0.92
TNF-alpha [ng/mL]				
- Pre-operative	0.39 [0.09; 0.6]	0.46 [0.21; 0.76]	0.33 [0.09; 0.7]	0.50
- ICU-admission	0.50 [0.21; 0.78]	0.5 [0.08; 0.81]	0.49 [0.27; 0.74]	0.90
- POD 1	0.35 [0.05; 0.55]	0.30 [0.11; 0.43]	0.42 [0.04; 0.60]	0.54

## Data Availability

The underlying data can be obtained upon request from the corresponding author.

## Abbreviations

The following abbreviations are used in this manuscript:

ARAS	Accelerated Recovery after Surgery
ASA	American Society of Anesthesiologists
CCI^®^	Comprehensive Complications Index
CO_2_	Carbon Dioxide
ELISA	Enzyme-linked immunosorbent assay
ERAS^®^	Enhanced Recovery after Surgery
FEV_1_	Forced expiratory volume in one second
ICU	Intensive care unit
IL	Interleukin
IQR	Interquartile range
TNF	Tumor necrosis factor
